# Impact of Mechanical Strain and Nicotinamide on RUNX2-Deficient Osteoblast Mimicking Cleidocranial Dysplasia

**DOI:** 10.3390/ijms242316581

**Published:** 2023-11-21

**Authors:** Agnes Schröder, Talia Örs, Ye-Oun Byeon, Fabian Cieplik, Peter Proff, Christian Kirschneck, Eva Paddenberg

**Affiliations:** 1 Department of Orthodontics, University Hospital Regensburg, 93053 Regensburg, Germany; talia.oers@stud.uni-regensburg.de (T.Ö.); ye-oun.byeon@stud.uni-regensburg.de (Y.-O.B.); peter.proff@ukr.de (P.P.); eva.paddenberg@ukr.de (E.P.); 2 Department of Conservative Dentistry and Periodontology, University Hospital Regensburg, 93053 Regensburg, Germany; fabian.cieplik@ukr.de; 3 Department of Orthodontics, University Hospital Bonn, 53127 Bonn, Germany; christian.kirschneck@uni-bonn.de

**Keywords:** cleidocranial dysplasia (CCD), orthodontic tooth movement, mechanical strain, bone remodeling

## Abstract

Cleidocranial dysplasia (CCD) is a rare genetic defect caused by a heterozygous mutation of runt-related transcription factor 2 (RUNX2), which is important for osteoblast and skeletal development. RUNX2-deficiency causes extra- and intra-oral malformations that often require orthodontic treatment. Nicotinamide (NAM) affects bone remodelling processes. As these are crucial for orthodontic therapy, NAM could improve orthodontic treatment in CCD patients. This study investigates the effect of NAM in control and RUNX2-deficient osteoblasts under mechanical strain mimicking orthodontic treatment. First, the optimal NAM concentration and the differences in the expression profile of control and RUNX2-deficient osteoblasts were determined. Subsequently, osteoblasts were exposed to tensile and compressive strain with and without NAM, and the expression of genes critically involved in bone remodelling was investigated. NAM increased the expression of bone remodelling genes. RUNX2-deficient osteoblasts expressed more receptor activator of NFkB ligand (RANKL) and interleukin-6 (IL6), but less colony-stimulating factor-1 (CSF1). Most of the positive effects of NAM on bone remodelling genes were impaired by mechanical loading. In conclusion, NAM stimulated osteoblast differentiation by increasing the expression of RUNX2 and regulated the expression of osteoclastogenic factors. However, the positive effects of NAM on bone metabolism were impaired by mechanical loading and RUNX2 deficiency.

## 1. Introduction

**Figure 1:**
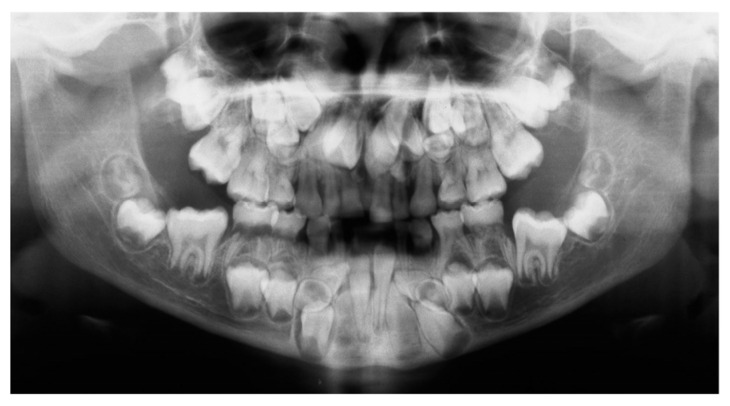
Orthopantomogram of a nearly 10-year-old female patient with Cleidocranial dysplasia. Dentitio tarda is present, the first molars (six-year molars) are not yet fully erupted. Supernumerary permanent teeth are present in the maxilla and in the mandible.

Dental anomalies usually lead to challenges in orthodontic treatment and often result in aesthetic and functional impairment in patients [1,2,3]. Cleidocranial dysplasia (CCD) is a rare congenital skeletal dysplasia, with an autosomal dominant pattern of inheritance and a prevalence of 1:1,000,000 [4]. Common clinical symptoms comprise extra- and intra-oral symptoms (Table 1) [5,6]. Dental-relevant symptoms include decreased eruption force of both dentitions, an altered eruption pattern of the teeth and hyperdontia (Figure 1; Table 1) [7]. The main cause of CCD is haploinsufficiency in the runt-related transcription factor 2 (*RUNX2*) gene [8,9]. *RUNX2* encodes an essential transcription factor for osteoblast differentiation and skeletal development [10]. Due to the dental symptoms, many CCD patients require orthodontic treatment (Figure 1) [11,12].

Orthodontic tooth movement is achieved by bone remodelling processes in the alveolar bone [13]. The application of an orthodontic force to the crown of a tooth creates pressure and tension zones in the periodontal ligament. Bone resorption by osteoclasts occurs in the pressure zones, while new bone is formed by osteoblasts in the tensile zones. Therefore, the success of orthodontic treatment is dependent on bone remodelling.

Bone remodelling is controlled by two major cell types: bone-forming osteoblasts and bone-resorbing osteoclasts. Osteoclast differentiation and activity is regulated via the expression of colony-stimulating factor 1 (CSF1), receptor activator of NFkB ligand (RANKL) and its decoy receptor, osteoprotegerin (OPG) [14,15]. During orthodontic treatment, mechanical stimuli increase the expression of inflammatory mediators like interleukin-6 (IL6) and tumor necrosis factor alpha (TNFα). Both cytokines enhance the secretion of RANKL by osteoblasts and periodontal ligament fibroblasts [13]. Osteoclast activity can be modulated by RUNX2. It increases the expression of OPG and inhibits the expression of RANKL [16].

Nicotinamide (NAM) impacts the expression of RUNX2, thereby affecting osteoclastogenesis [17]. NAM is absorbed into cells in the form of nicotinamide adenine dinucleotide (NAD^+^) and nicotinamide adenine dinucleotide phosphate (NADP^+^), which are important cofactors in many cellular processes [18]. As it was demonstrated that NAM impacts on delayed tooth eruption in RUNX2-deficient mice, it could be a therapeutic tool for the treatment of dental anomalies in patients with CCD.

To date, there is little evidence about the effect of mechanical loading itself on RUNX2-deficient osteoblasts. As NAM is discussed as a therapeutical agent in the treatment of CCD, there should be studies on the impact of mechanical strain on the effects of NAM. This study investigates the influence of heterozygous RUNX2 mutations and the impact of NAM on bone remodeling genes during mechanical strain occurring during orthodontic tooth movement. We hypothesize that RUNX2 deficiency and NAM both have an effect on the expression profile of osteoblasts, perhaps leading to an impairment in orthodontic treatment.

## 2. Results

### 2.1. Impact of Different Nicotinamide (NAM) Concentrations on Control Osteoblasts

**Figure 2:**
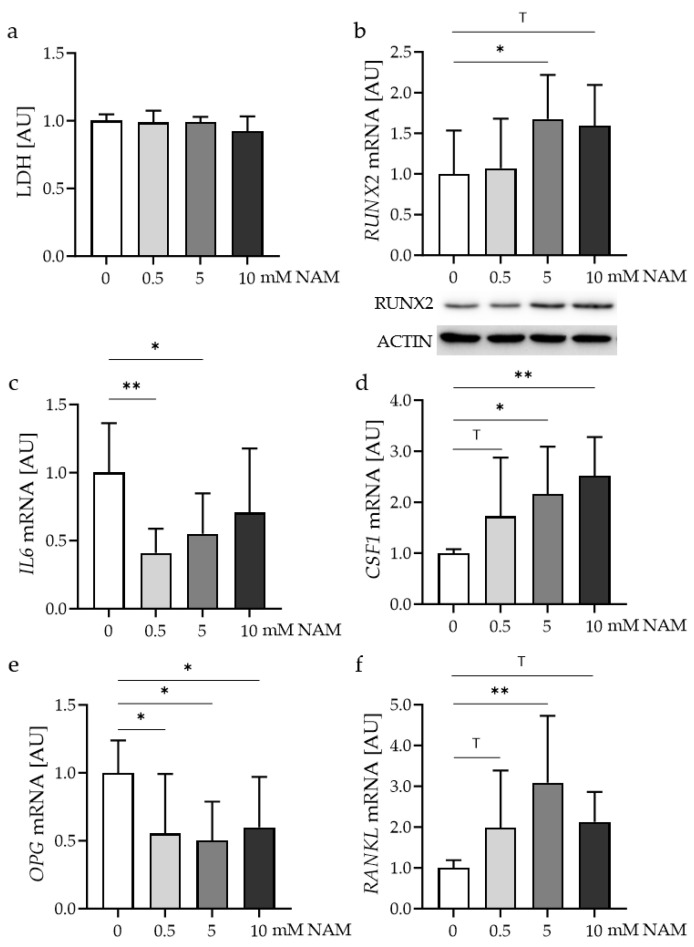
Effects of different NAM concentrations on lactate dehydrogenase (LDH) release (**a**) and expression of *RUNX2* (**b**), *IL6* (**c**), *CSF1* (**d**) *OPG* (**e**) and *RANKL* mRNA (**f**) in human control osteoblasts; *n* ≥ 8. Statistics: ANOVA with Holm-Šídák’s multiple comparisons test. ^T^ *p* < 0.10, * *p* < 0.05, ** *p* < 0.01.

First, the effect of different nicotinamide (NAM) concentrations on human control osteoblasts was investigated. All tested NAM concentrations had no cytotoxic effects on the cells, as lactate dehydrogenase (LDH) release remained unaffected (Figure 2a). A concentration of 5 or 10 mM NAM increased the gene and protein expression of runt-related transcription factor 2 (RUNX2; Figure 2b). The gene expression of interleukin-6 (*IL6*) was reduced with 0.5 and 5 mM NAM (Figure 2c). The expression of colony-stimulating factor-1 (*CSF1*) was enhanced with 5 mM and 10 mM NAM (Figure 2d). A significant reduction was observed for osteoprotegerin (*OPG*) mRNA with all tested NAM concentrations (Figure 2e). The receptor activator of NFkB ligand (*RANKL*) mRNA was significantly increased with 5 mM NAM, while there was a noticeable trend for 0.5 mM and 10 mM NAM (Figure 2f). A concentration of 5 mM NAM shifted the RANKL/OPG ratio towards RANKL, indicating increased osteoclast differentiation. Therefore, the following experiments were performed with a concentration of 5 mM NAM.

### 2.2. Impact of NAM on Control and RUNX2-Deficient Osteoblasts

**Figure 3:**
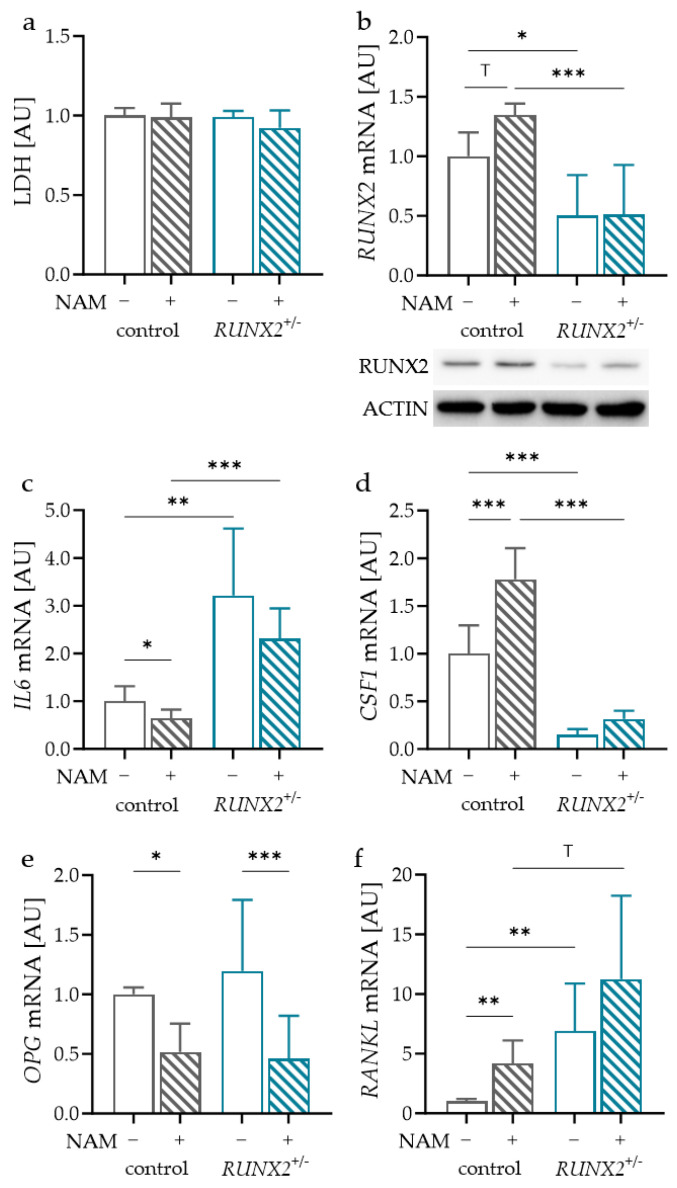
Effects of NAM and RUNX2 deficiency on lactate dehydrogenase (LDH) release (**a**) and expression of *RUNX2* (**b**), *IL6* (**c**), *CSF1* (**d**) *OPG* (**e**) and *RANKL* mRNA (**f**) in control or RUNX2-deficient (*RUNX2^+/−^*) osteoblasts; *n* ≥ 5. Statistics: LDH, *RUNX2* mRNA, *CSF1* mRNA, *OPG* mRNA: ANOVA with Holm-Šídák’s multiple comparisons test; *IL6* mRNA, *RANKL* mRNA: Welch-corrected ANOVA with Dunnett’s T3 multiple comparisons test ^T^ *p* < 0.10, * *p* < 0.05, ** *p* < 0.01, *** *p* < 0.001.

Next, the effect of NAM on the expression of bone remodeling genes in control and RUNX2-deficient (RUNX2^+/−^) osteoblasts was obtained. In both osteoblast cell lines, NAM had no effect on LDH release, indicating no cytotoxic effects (Figure 3a). To ensure RUNX2 deficiency, gene and protein expression were determined. RUNX2-deficient osteoblasts showed reduced RUNX2 gene and protein expression with and without NAM treatment (Figure 3b). The addition of NAM increased RUNX2 gene and protein expression in the control osteoblasts (Figure 3b). In RUNX2-deficient osteoblasts, only the effect of NAM on RUNX2 protein expression was visible (Figure 3b).

*IL6* mRNA expression was reduced in the control osteoblasts with NAM (Figure 3c). In *RUNX2^+/−^* osteoblasts, there was an increase in *IL6* gene expression with and without NAM treatment compared to the control osteoblasts. This indicates a regulatory role of RUNX2 in *IL6* mRNA expression. There was no reducing effect of NAM on *IL6* gene expression in RUNX2-deficient osteoblasts (Figure 3c). The gene expression of *CSF1* was enhanced by NAM in the control osteoblasts (Figure 3d). RUNX2 deficiency reduced *CSF1* mRNA with and without NAM treatment. Like *IL6* mRNA, the effect of NAM was no longer detectable in *RUNX2^+/−^* osteoblasts (Figure 3d). A significant reduction was observed for *OPG* mRNA with NAM in the control and *RUNX2*^+/−^ osteoblasts (Figure 3e). RUNX2 deficiency had no effect on *OPG* gene expression (Figure 3e). NAM treatment significantly increased *RANKL* mRNA in the control osteoblasts (Figure 3f). In *RUNX2*^+/−^ osteoblasts, *RANKL* gene expression was elevated with no additional effect of NAM (Figure 3f). RUNX2 in osteoblasts seemed to play a regulatory role in *IL6*, *CSF1* and *RANKL* gene expression, thereby affecting bone remodeling processes. RUNX2 deficiency impaired the effect of NAM for all investigated genes except *OPG*.

### 2.3. Impact of NAM on Control and RUNX2-Deficient Osteoblasts during Tensile Strain

**Figure 4:**
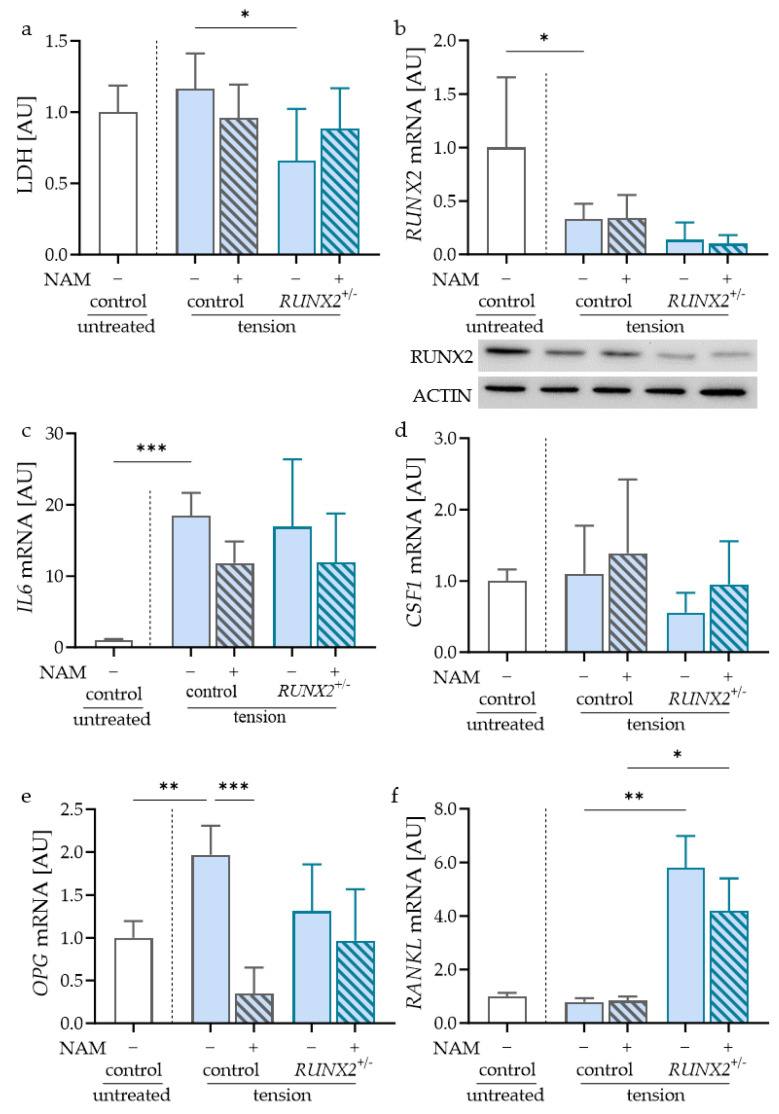
Effects of NAM and RUNX2 deficiency on LDH release (**a**) and expression of *RUNX2* (**b**), *IL6* (**c**), *CSF1* (**d**) *OPG* (**e**) and *RANKL* mRNA (**f**) in osteoblasts during tensile strain; *n* = 5. Statistics: ANOVA with Holm-Šídák’s multiple comparisons test expect for *RANKL* mRNA: Welch-corrected ANOVA with Dunnett’s T3 multiple comparisons test * *p* < 0.05, ** *p* < 0.01, *** *p* < 0.001.

The application of orthodontic force led to pressure and tensile zones in the periodontal ligament. To investigate the possible effects of orthodontic treatment on NAM treatment in control and RUNX2-deficient osteoblasts, the cells were first exposed to tensile strain. No effects on LDH release, as an indicator of cytotoxicity, were observed for NAM or RUNX2 deficiency after stretching (Figure 4a). As expected, RUNX2 expression was lower in *RUNX2*^+/−^ osteoblasts. In control osteoblasts, *RUNX2* mRNA was reduced in reaction to tensile strain (Figure 4b). Stretching the control osteoblasts prevented the effect of NAM on RUNX2 gene or protein expression (Figure 4b). *IL6* gene expression was elevated in the control osteoblasts in reaction to tension (Figure 4c). After the application of tensile strain, there was no significant effect of NAM on *IL6* mRNA detected in the control or RUNX2-deficient osteoblasts (Figure 4c). *IL6* mRNA expression in *RUNX2*^+/−^ osteoblasts was similar to the control osteoblasts after stretching (Figure 4c). For *CSF1* mRNA, no effect of NAM or RUNX2 deficiency was detected after tensile strain (Figure 4d). *OPG* gene expression was increased in the control osteoblasts in reaction to tensile strain (Figure 4e). NAM reduced this inductive effect of stretching in the control osteoblasts, while there was no effect on RUNX2-deficient osteoblasts under tensile strain (Figure 4e). Stretching had no effect on *RANKL* gene expression in the control cells. NAM failed to induce *RANKL* mRNA in the control osteoblasts exposed to tension (Figure 4f). The increased expression of *RANKL* mRNA was observed in *RUNX2*^+/−^ osteoblasts with and without NAM treatment (Figure 4f). NAM itself had no significant effect on *RANKL* gene expression in RUNX2-deficient osteoblasts after tensile strain. These data showed the impact of tensile strain on the effect of NAM, not only on the control but also on RUNX2-deficient osteoblasts.

### 2.4. Impact of NAM on Control and RUNX2-Deficient Osteoblasts during Compressive Strain

**Figure 5:**
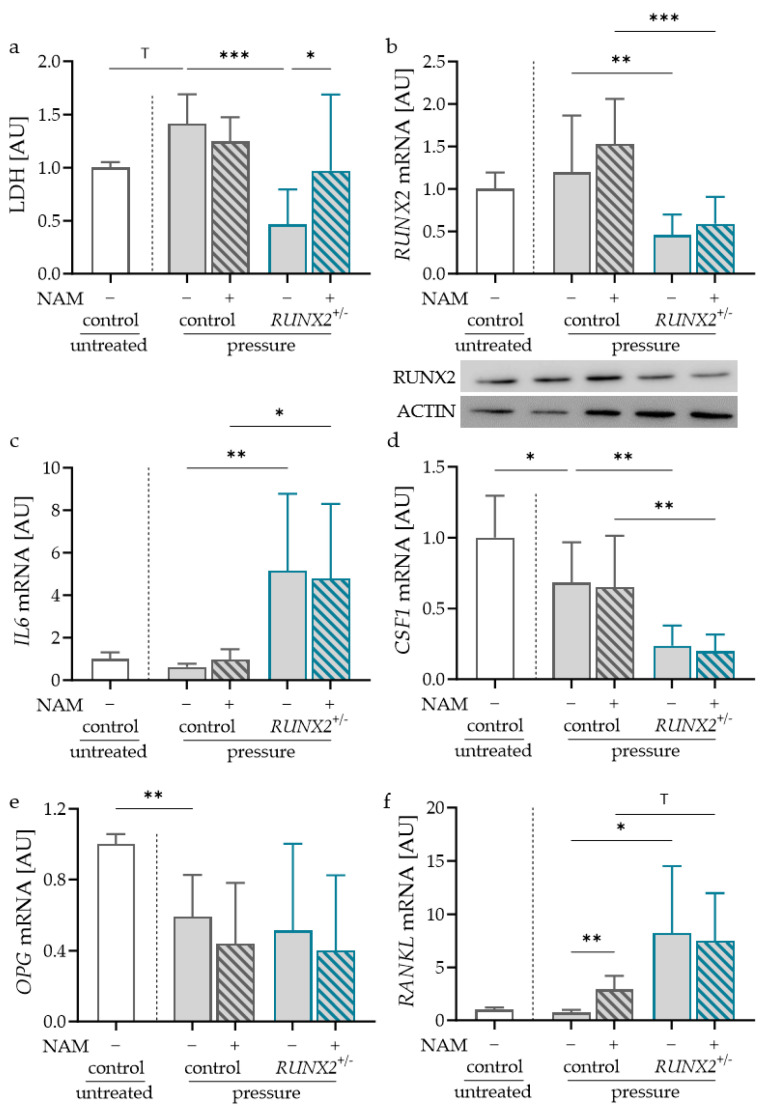
Effects of NAM and RUNX2 deficiency on LDH release (**a**) and expression of *RUNX2* (**b**), *IL6* (**c**), *CSF1* (**d**) *OPG* (**e**) and *RANKL* mRNA (**f**) in osteoblasts during compressive strain; *n* ≥ 7. ANOVA with Holm-Šídák’s multiple comparisons test expect for *OPG* and *RANKL* mRNA: Welch-corrected ANOVA with Dunnett´s T3 multiple comparisons test ^T^ *p* < 0.10, * *p* < 0.05,** *p* < 0.01, *** *p* < 0.001.

Next to tensile forces, cells in the periodontal ligament were also exposed to compressive strain during orthodontic treatment. Therefore, the control and *RUNX2*^+/−^ osteoblasts were exposed to compressive strain to investigate the possible impact of this mechanical loading on the effects of NAM. With compressive strain, there was a reduction in LDH release in RUNX2-deficient osteoblasts compared to the control osteoblasts (Figure 5a). Treatment with NAM, in combination with pressure application, increased LDH release in *RUNX2*^+/−^ osteoblasts, indicating a cytotoxic effect (Figure 5a).

Pressure application itself showed no effect on the expression of *RUNX2* mRNA in the control osteoblasts (Figure 5b). Similar to tensile strain, the effect of NAM was impaired in the control osteoblasts after exposure to compressive strain. As expected, *RUNX2* mRNA was reduced in *RUNX2*^+/−^ osteoblasts (Figure 5b). After pressure application, NAM had no effect on RUNX2 expression in RUNX2-deficient osteoblasts. The gene expression of *IL6* was not affected by pressure application in the control osteoblasts. The inhibitory effect of NAM on *IL6* gene expression was abolished in the control osteoblasts exposed to compressive strain (Figure 5c). After pressure application, *IL6* mRNA was increased in RUNX2-deficient cells with and without NAM treatment compared to the control osteoblasts (Figure 5c). *CSF1* mRNA was reduced in the control osteoblasts after compressive strain (Figure 5d). Again, NAM had no longer an effect on *CSF1* mRNA expression in control cells (Figure 5d). As seen before, without compressive strain, a deficiency in RUNX2 reduced *CSF1* gene expression (Figure 5d). For *OPG* mRNA expression, there was a reduction in the control osteoblasts after exposure to detectable compressive strain (Figure 5e). With this mechanical loading, NAM failed to affect *OPG* mRNA in the control and RUNX2-deficient osteoblasts. For *RANKL* mRNA expression, there was still an induction observed after NAM treatment in the control osteoblasts (Figure 5f). In *RUNX2*^+/−^ osteoblasts, *RANKL* gene expression was increased with no additional effect of NAM (Figure 5f). Similar to tensile strain, pressure application impacted the effect of NAM in control and RUNX2-deficient osteoblasts. Of note, there were differences between tensile and compressive strain in the effects on the cells.

## 3. Discussion

The aims of this study were to (1) investigate the impact of nicotinamide (NAM) on control and runt-related transcription factor 2 (RUNX2)-deficient osteoblasts and (2) to identify possible effects of mechanical strains occurring during orthodontic tooth movement on the effects of NAM on bone remodeling genes. RUNX2-deficient osteoblasts were used in this study to mimic cleidocranial dysplasia (CCD).

In line with previous studies, there was a dose-dependent effect of NAM on genes involved in bone remodeling, like RUNX2, interleukin-6 (*IL6*), colony-stimulating factor-1 (*CSF1*), osteoprotegerin (*OPG*) and receptor activator of NFkB ligand (*RANKL*) [17]. Yoon et al. examined the effects of NAM on RUNX2 expression in a mouse model. After the application of NAM, RUNX2 protein expression increased in RUNX2-deficient osteoblasts to the level of the untreated control osteoblasts [17]. NAM stabilized RUNX2 by inhibiting sirtuin 2 (SIRT2), which decreased the stability and activity of RUNX2 through deacetylation [17]. In CCD patients, reduced RUNX2 expression was associated with impaired osteogenic capabilities. Liu et al. restored RUNX2 expression in CCD cells using a lentivirus expressing wildtype RUNX2. This increased *RUNX2* mRNA and restored the osteogenic capability of the infected CCD cells [19].

The expression of the inflammatory gene IL6 was increased in the periodontal ligament after orthodontic treatment [20,21,22]. Here, IL6 was significantly higher in the absence of RUNX2 or after tensile strain in the control osteoblasts. In contrast, compressive strain had no effect on *IL6* gene expression in the control osteoblasts. The application of NAM reduced IL6 expression in the control osteoblasts. Contrary to the data on osteoblasts, periodontal ligament fibroblasts increased IL6 after compressive strain, while there was a reduction after tensile strain [20,23]. Macrophages reacted to both mechanical loading protocols with an induction of IL6 expression [21]. IL6 is critically involved in osteoclast recruitment [24], but it could also have both inhibitory and stimulatory effects on osteoclastogenesis, and thus could promote bone formation or degradation [25]. Therefore, the observed impairment of IL6 due to RUNX2 deficiency, NAM application or tensile loading could affect bone remodeling.

*CSF1* is a growth factor required for osteoclast differentiation, maturation and survival. *CSF1* released by osteoblasts stimulated the proliferation of osteoclast precursors via the c-fms receptor and, in combination with *RANKL*, led to the formation of mature osteoclasts [26]. RUNX2 directly bound to the promoter region of *CSF1* [17]. In accordance with prior research, we observed enhanced *CSF1* expression after NAM treatment [17]. Due to the deficiency in RUNX2, there was a decrease in *CSF1* mRNA expression and a concomitant increase in *RANKL* expression. A reduction in *CSF1* expression might cause a decrease in osteoclast activity and thus reduced bone resorption. In contrast, a measured increase in *RANKL* promoted osteoclast formation and activity and thus contributed to bone resorption [27,28]. Yoon et al. described an increased RANKL/OPG ratio upon administration of NAM in *RUNX2*^+/−^ mice leading to increased bone remodeling. OPG acted as a RANKL decoy receptor, which prevented the interaction of RANKL with the membrane-bound RANK receptor on osteoclast precursor cells [27,28]. In line with this, a significant increase in RUNX2 expression and RANKL/OPG ratio was measured in control cells after the application of NAM in our study. The experimental adenoviral introduction of RUNX2 into a RUNX2-deficient cell line induced RANKL expression, suppressed OPG expression and thus restored osteoclast differentiation. This suggests that RUNX2 promotes osteoclast differentiation by inducing RANKL and inhibiting OPG [29]. Similar results by Yoda et al. also suggested that RUNX2 was involved in osteoclastogenesis via activation of RANKL expression and its receptor [30]. Since an increase in RANKL was also observed in RUNX2-deficient osteoblasts, RANKL levels alone do not appear to be suitable as an indicator of decreased bone resorption. In general, it was seen that an imbalance between CSF1 and RANKL led to impaired bone metabolism and thus decreased osteoclastogenesis. According to Yoon, it could be suggested that CSF1 may be a key regulator in the impairment of osteoclastogenesis in patients with CCD [17].

This study had several limitations that should be mentioned. As human osteoblasts were used, individual effects could be present. For this reason, similar experiments should be repeated with established osteoblastic cell lines. In this study, osteoblasts were subjected to mechanical strain for short time periods corresponding to experimental setups with macrophages [21]. Other cells, like periodontal ligament fibroblasts, need longer to react to orthodontic strains [20]. Therefore, experiments with longer time periods should be performed. Furthermore, orthodontic tooth movement is a multicellular process, in which osteoblasts, osteoclasts, immune cells and periodontal ligament fibroblasts play crucial roles. These cell types could also be influenced by NAM in their response to orthodontic forces. Thus, further studies are needed here.

This study showed that NAM had positive effects on bone remodeling in control and RUNX2-deficient osteoblasts. It could, therefore, have positive effects on CCD patients in regard of bone remodeling. However, we also showed that these positive effects of NAM were impaired by mechanical loading. The effects also depended on the loading protocols and were different for tensile and compressive forces. Therefore, further experiments are needed to investigate the effects of mechanical strains occurring during orthodontic tooth movement on these NAM effects.

NAM was shown to promote osteoblast differentiation by increasing the protein level of RUNX2. This increased the expression of osteoclastogenic genes. In RUNX2-deficient cells, the application of NAM affected the impaired osteoclastogenesis. Mechanical loading interfered with the effects of NAM on bone remodeling genes. Therefore, further studies are needed to explore a possible therapy for CCD patients with NAM during orthodontic therapy.

## 4. Materials and Methods

### 4.1. Cultivation of Human Osteoblasts

#### 4.1.1. General Cell Culture Conditions

The control osteoblasts used in this study were obtained from the nasal septum of a healthy patient. The heterozygous RUNX2 mutant osteoblasts were obtained from a cleidocranial dysplasia (CCD) patient (12-170-0150) [31]. The cells were cultivated in Dulbecco’s Modified Eagle’s High Glucose (DMEM, D5671, Sigma-Aldrich, St. Louis, MO, USA), supplemented with 10% fetal bovine serum (FBS; P30-3302, PAN-Biotech, Germany), 1% Antibiotic/Antimycotic Solution (AA; A5955, Sigma-Aldrich) and 1% L-glutamine (G7513, Sigma-Aldrich) at 37 °C and 5% CO_2_. The cell number was determined using the Beckmann Coulter Counter Z2. Approximately 130,000 osteoblasts per mL were seeded for the experiments, either in 24-well plates (300 µL) for RNA isolation or 6-well plates (1.2 mL) for protein isolation in Minimum Essential Medium (αMEM, RNBK3712, Sigma-Aldrich) with 10% FBS (P30-3302, PAN-Biotech), 1% AA (A5955, Sigma-Aldrich) and 1% L-glutamine (G7513, Sigma-Aldrich).

#### 4.1.2. Experimental Setup to Determine the Optimal Nicotinamide (NAM) Concentration

**Figure 6:**
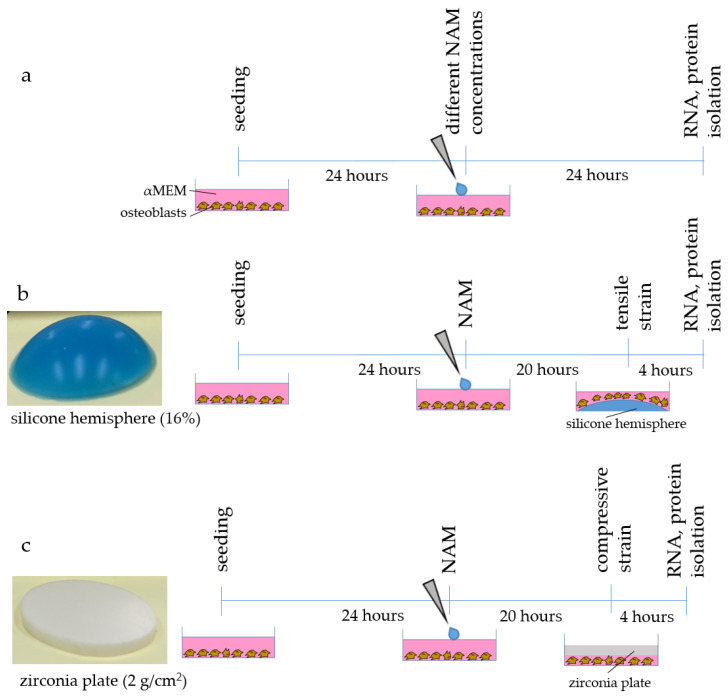
Schematic representation of the experimental setup to determine the optimal NAM concentration (**a**), as well as application of tensile (**b**) and compressive strain (**c**).

The control osteoblasts were seeded as described above. One day after seeding, 0.5 mM, 5 mM, or 10 mM nicotinamide (NAM, 7340, Sigma-Aldrich) were added to the osteoblasts. The control samples remained without any NAM addition. The cells were incubated for an additional 24 h (Figure 6a). After the incubation period, the cells were harvested, and the RNA and proteins were isolated.

#### 4.1.3. Experimental Setup for Tensile Strain

For tensile strain, the osteoblasts were seeded onto 6-well BioFlex plates (BF-3001C, Dunn Labortechnik, Asbach, Germany). The application of 5 mM NAM (7340, Sigma-Aldrich) was performed for at least 24 h. Twenty hours after the addition of NAM, a silicone hemisphere (16%) was inserted outside the flexible well membrane and the cells were stretched for a further four hours (Figure 6b) [23].

#### 4.1.4. Experimental Setup for Compressive Strain

One day after seeding, the osteoblasts were either left untreated or 5 mM NAM (7340, Sigma-Aldrich) were added to the cells for a total of 24 h. Four hours before the end of the experiment, zirconia plates (2 g/cm^2^) were placed in the corresponding wells (Figure 6c) [32]. Before this, the plates were incubated in the corresponding medium for 10 min. After four hours of incubation, the RNA and proteins were isolated.

### 4.2. Determination of Cytotoxicity with the Lactate Dehydrogenase (LDH) Test

An LDH assay was performed to assess cytotoxicity. An LDH test (4744926001, Sigma-Aldrich) was used according to the manufacturer’s protocol. A photometric measurement of absorbance was performed using Multiscan Go (14142550, Thermo Scientific, Waltham, MA, USA).

### 4.3. RNA Analysis

#### 4.3.1. RNA Isolation

After removal of the medium, the cells were directly lysed in the wells using 250 µL trizol reagent (R6830-01, VWR, Radnor, PA, USA) and transferred to a reaction tube. Then, 100 µL chloroform (1916276, Fisher Chemical, Hampton, NH, USA) was added and the samples were vortexed for 30 s. After incubation for 15 min on ice, the samples were centrifuged at 13,000 rpm at 4 °C for 15 min. The aqueous supernatant was carefully transferred to a new tube with 500 µL ice cold isopropanol (20J234012, VWR Chemical, Radnor, PA, USA). This was followed by incubation for at least 24 h at −80 °C. After thawing, the samples were centrifuged for 30 min at 13,000 rpm at 4 °C. The supernatant was removed and the pellet was washed twice with 80% ethanol. The samples were dried for 30 min and the pellets were resolved in nuclease-free H_2_O_dd_ (1089F, Biochrom, Holliston, MA, USA). Finally, the RNA concentration was measured in a nanophotometer (N60, Implen, Westlake Village, CA, USA).

#### 4.3.2. copyDNA (cDNA) Synthesis

For cDNA synthesis, equal amounts of RNA were mixed with a master mix to minimize variations in a total volume of 10 µL. The master mix was composed of 0.5 µL M-MLV reverse transcriptase (M1705, Promega, Madison, WI, USA), 0.5 µL dNTPs (L785. 2, Carl Roth, Karlsruhe, Germany), 0.5 µL oligo(dT)18 primer (SO132, Thermo Fisher), 0.5 µL random hexamer primer (SO142, Thermo Fisher), 0.5 µL RiboLock Rnase inhibitor (EO0382, Thermo Fisher) and 2 µL M-MLV RT buffer (M531A, Promega). The samples were incubated at 37 °C for one hour, followed by incubation for 2 min at 95 °C in a thermal cycler (Biometra Tone 96G, Analytik Jena, Jena, Germany).

#### 4.3.3. Quantitative Polymerase Chain Reaction (qPCR)

To determine the expression of the investigated genes, an individual primer mix was prepared using specific primer pairs (Table 2). This consisted of 0.25 µL forward primer, 0.25 µL reverse primer, 5 µL Luna Universal qPCR Mix (10134796, New England BioLabs, Ipswich, MA, USA) and 3 µL RNase-free water (1089F, Biochrom) per well. Following preparation, 1.5 µL of the cDNA was mixed with 8.5 µL of the primer mix in a 96-well plate (712282, Biozym, Hessisch Oldendorf, Germany). All samples were pipetted as duplicates. The plates were taped with foil (712350, Biozym) and briefly centrifuged. qPCR was performed in the master cycler realplex 2 (Eppendorf, Hamburg, Germany). After heating to 95 °C for 5 min, 45 repeat cycles (95 °C for 10 s, 60 °C for 8 s, 72 °C for 8 s) were conducted. A combination of *GAPDH* and *TBP* were used as reference genes (Table 2). Relative gene expression was calculated using the formula 2^−ΔCT^, where ΔCT was the difference between the CT value of the target gene and the geometric mean of *GAPDH/TBP* [33,34].

### 4.4. Protein Analysis

#### 4.4.1. Protein Isolation and Determination of Protein Concentration

After the removal of the cell culture supernatant, 100 µL CellLyticM (C2979, Sigma-Aldrich), supplemented with 1 µL protease inhibitor (87786, Thermo Fisher), was added to the adherent cells. The cells were detached using a cell scraper (83.1830, Sarstedt, Nümbrecht, Germany), transferred to reaction tubes and incubated on ice for 15 min. The samples were vortexed every 5 min. Subsequently, the samples were centrifuged at 13,000 rpm for 10 min at 4 °C and the supernatant was pipetted into new reaction tubes. The cell pellet was then discarded. A Bradford assay was performed to determine the protein concentration (K015.3, Carl Roth).

#### 4.4.2. Polyacrylamide (PAA) Gel Electrophoresis and Western Blot

Equal protein concentrations were mixed with 6x loading buffer (3.75 mL Tris (T1503, Sigma-Aldrich), 3 mL glycerol (3783.1, Carl Roth), 1.2 g sodium dodecyl sulfate (8029.1, Carl Roth), 0.06 g bromophenol blue (B-5525, Sigma-Aldrich) and 15 mg/mL dithiotreitol (6908.1, Carl Roth)) and heated to 70 °C for 7 min. The samples were homogenized and centrifuged. Proteins were separated on 12% PAA gels and transferred to polyvinylidenfluoride membranes (T830.1, Carl Roth). After blotting, the membranes were blocked with 5% milk (T145.3, Carl Roth) in tris-buffered saline (50 mM Tris (T1503, Sigma-Aldrich); 150 mM sodium chloride (9265.2; Carl Roth)) with 1% Tween-20 (9127.1; Carl Roth) (TBS-T) and incubated in the primary antibody solution (RUNX2 (2534665, MyBiosource) or ACTIN (CA2066, Sigma-Aldrich) diluted 1:3000 in 5% milk (T145.3, Carl Roth)) in TBS-T overnight with agitation. The membranes were washed three times with TBS-T for 10 min each. This was followed by incubation with the secondary horseradish peroxidase-coupled antibody (611-1303, RockLand, Rockland, NY, USA), diluted 1:5000 in 5% milk (T145.3, Carl Roth) in TBS-T for one hour. After washing with TBS-T three times, Luminata Crescendo (WBLUR0100, Sigma-Aldrich) was pipetted onto the membrane. A VWR Genoplex was used for the digitalization of the signal.

## 5. Conclusions

In conclusion, NAM stimulates osteoblast differentiation by increasing the protein level of RUNX2 and regulating the expression of osteoclastogenic factors in osteoblasts. The application of NAM normalizes the decreased osteoclastogenesis in CCD patients with a RUNX2 deficit. However, mechanical strain impairs the effect of NAM on osteoblasts. Therefore, further studies are needed to make more precise statements on whether and how the treatment of CCD patients with NAM and mechanical stress is possible. A long-term study would also be desirable to assess the long-term effect of NAM on control and RUNX2-deficient cells.

## Figures and Tables

**Table 1:** Summary of general and dental clinical symptoms of CCD [5,6].

General Clinical Symptoms	Dental Symptoms
Abnormal height	Hyperdontia
Open fontanelles	Supernumerary tooth germs
Bone-related problems	Wide spacing in the lower incisor area
Clavicular hypoplasia	Parallel-sided ascending rami
Congenital hip luxation	Altered eruption pattern
Joint hypermobility	Gingival cysts

**Table 2:** Reference and target gene primers used for pPCR.

Gene	Gene Name	Forward Primer	Reverse Primer
*CSF1*	Colony-stimulating factor 1	TGAGACACCTCTCCAGTTGCTG	GCAATCAGGCTTGGTCACCACA
*GAPDH*	Glycerinaldehyde-3-phosphate-dehydrogenase	TGCCCTCAACGACCACTTTG	CCACCACCCTGTTGCTGTAG
*IL6*	Interleukin-6	TGGCAGAAAACAACCTGAACC	CCTCAAACTCCAAAAGACCAGTG
*OPG*	Osteoprotegerin	TGTCTTTGGTCTCCTGCTAACTC	CCTGAAGAATGCCTCCTCACAC
*RUNX2*	Runt-related transcription factor 2	CAGTAGATGGACCTCGGGAAC	TGAGGCGGTCAGAGAACAAAC
*RANKL*	Receptor activator of NFkB ligand	ATACCCTGATGAAAGGAGGA	GGGGCTCAATCTATATCTCG
*TBP*	TATA binding protein	CGGCTGTTTAACTTCGCTTCC	TGGGTTATCTTCACACGCCAAG

## Data Availability

The data underlying this article will be shared upon reasonable request to the corresponding author.
